# IBD-Associated *TL1A* Gene (*TNFSF15*) Haplotypes Determine Increased Expression of TL1A Protein

**DOI:** 10.1371/journal.pone.0004719

**Published:** 2009-03-05

**Authors:** Kathrin S. Michelsen, Lisa S. Thomas, Kent D. Taylor, Qi T. Yu, Ling Mei, Carol J. Landers, Carrie Derkowski, Dermot P. B. McGovern, Jerome I. Rotter, Stephan R. Targan

**Affiliations:** 1 Inflammatory Bowel Disease Center & Immunobiology Research Institute, Cedars-Sinai Medical Center, Los Angeles, California, United States of America; 2 Medical Genetics Institute, Cedars-Sinai Medical Center, Los Angeles, California, United States of America; HelmholtzZentrum München, Germany

## Abstract

**Background:**

The recently identified member of the TNF superfamily TL1A (*TNFSF15*) increases IFN-γ production by T cells in peripheral and mucosal CCR9+ T cells. TL1A and its receptor DR3 are up-regulated during chronic intestinal inflammation in ulcerative colitis and Crohn's disease (CD). *TL1A* gene haplotypes increase CD susceptibility in Japanese, European, and US cohorts.

**Methodology and Principal Findings:**

Here we report that the presence of *TL1A* gene haplotype B increases risk in Jewish CD patients with antibody titers for the E. coli outer membrane porin C (OmpC+) (Haplotype B frequency in Jewish CD patients: 24.9% for OmpC negative and 41.9% for OmpC positive patients, respectively, P≤0.001). CD14+ monocytes isolated from Jewish OmpC+ patients homozygous for *TL1A* gene haplotype B express higher levels of TL1A in response to FcγR stimulation, a known inducing pathway of TL1A, as measured by ELISA. Furthermore, the membrane expression of TL1A is increased on peripheral monocytes from Jewish but not non-Jewish CD patients with the risk haplotype.

**Conclusions and Significance:**

These findings suggest that *TL1A* gene variation exacerbates induction of TL1A in response to FcγR stimulation in Jewish CD patients and this may lead to chronic intestinal inflammation via overwhelming T cell responses. Thus, TL1A may provide an important target for therapeutic intervention in this subgroup of IBD patients.

## Introduction

TL1A, a recently identified member of the TNF superfamily, increases IL-2 response by anti-CD3/CD28-stimulated T cells [1]. Furthermore, we and others have shown that TL1A synergizes with IL-12 and IL-18 to augment IFN-γ release in human T and NK cells and biases T cell differentiation towards a T_H_1 phenotype [2], [3], [4]. TL1A expression is increased in inflamed tissue of colon and small bowel of CD patients and colocalizes to macrophages and T cells [2], [5]. In particular, lamina propria, but also peripheral CD4+CCR9+ T cells, constitutively express membrane TL1A and are especially sensitive to TL1A stimulation [3], [4]. In murine models of ileitis, TL1A is mainly expressed on lamina propria dendritic cells [6].

We have recently demonstrated that TL1A is produced by antigen-presenting cells, e.g. monocytes and dendritic cells, in response to FcγR signaling but not in response to Toll-like receptor agonists or pro-inflammatory cytokines [7]. Stimulation with Immune Complexes (IC) leads to the expression of both membrane and secreted TL1A [1], [7]. Neutralizing TL1A antibodies prevent and treat colitis in a murine model of chronic colitis by affecting both T_H_1 and T_H_17 responses, suggesting that TL1A is a central regulator of intestinal inflammation during colitis [8]. In addition, it has been demonstrated recently that TL1A also plays an important role in the pathogenesis of other inflammatory diseases, such as Experimental Autoimmune Encephalomyelitis (EAE) and allergic lung inflammation [9], [10], [11].

The first genome-wide association study of CD provided evidence that variation in *TNFSF15*, the *TL1A* gene, contribute to CD in Japanese and both CD and ulcerative colitis in the British population [12], [13]. Haplotypes composed of 5 *TNFSF15* SNPs were observed to confer significant CD risk (*haplotype A*) and protection (*haplotype B*), using both case/control and family-based study designs [12], [13], [14]. We have also observed a protective association between CD and the same *haplotype B* in a Los Angeles based cohort [15]. Stratification on Ashkenazi Jewish ethnicity suggested that *haplotype B* may have a different effect on CD susceptibility in the Jewish and non-Jewish populations. In contrast to the protective association seen in non-Jews, the opposite trend towards a risk association with *haplotype B* was observed in Ashkenazi Jews [15]. Similar observation of differential genetic risk association in diverse ethnic groups have been made in CD, in ulcerative colitis and other gentically complex diseases including schizophrenia and asthma [16], [17], [18], [19], [20], [21], [22]. Jewish CD patients carrying the *TNFSF15 haplotype B* were more likely to have more severe CD, as evidenced by a higher rate of surgery [15] and by the expression of antibody responses to microbial antigens, including the *E. coli* outer membrane porin C (OmpC+) [23], [24]. To date, no functional basis for the relationship between *TNFSF15* variation and disease severity in CD patients has been shown.

In order to determine the functional consequences of *TNFSF15* genetic variation, we have identified subjects for immunological studies based on *TNFSF15 haplotypes*. Here we show that, in Jewish CD patients with seropositivity for OmpC, the TL1A risk haplotype *TNFSF15 haplotype B* is associated with higher TL1A expression upon stimulation of FcγR. Furthermore, Jewish but not non-Jewish CD patients with the *TNFSF15* risk *haplotype B* have a higher baseline expression of TL1A on peripheral monocytes, suggesting a higher baseline capacity for T cell stimulation. Collectively, our data define a role for *TNFSF15* genetic variation in determining disease severity in Jewish CD patients, and support the concept that TL1A is a novel interventional target, at least for the subgroup of Jewish, OmpC+, CD patients.

## Methods

### Human subjects

We collected peripheral blood from randomly selected patients attending the IBD center at Cedars-Sinai Medical Center who had previously been diagnosed with CD according to standard clinical, endoscopic, radiological, and histological findings. Written informed consent was obtained from all patients. Procedures were approved by the Institutional Review Board of Cedars-Sinai Medical Center (IRB number 3358 and 2673). The patient's demographics, diagnoses and medications at time of sample collection are provided in Table 1. The medications were equivalent in the different groups. Jewish ethnicity was defined as previously described by one or more grandparents of Ashkenazi Jewish descent [25], [26]. Controls were matched for ethnicity and were usually spouses of CD patients.

**Table 1:** Patient's demographic, diagnoses, medications.

Ethnicity	tnfsf15 haplotype_a	tnfsf15 haplotype_b	OMPC status	Disease Activity	Current IBD Medications
Jewish	A	A	Positive	Inactive	Asacol
Jewish	A	A	Positive	Inactive	Asacol, Purinethol
Jewish	A	A	Positive	Inactive	Asacol, 6-MP
Jewish	A	A	Positive	Inactive	Asacol, Remicade every 10 wks
Jewish	A	not B	Positive		Remicade
Jewish	A	A	Positive	Active	Remicade
Jewish	A	A	Positive	Inactive	Pentasa, 6-MP
Jewish	not A	B	Positive	Inactive	Asacol. 6-MP
Jewish	not A	B	Positive	Inactive	Budesonide
Jewish	not A	B	Positive	Active	Remicade monthly
Jewish	B	B	Positive	Active	Entocort
Jewish	B	B	Positive	Inactive	6-MP, Remicade every 3 months
Jewish	B	B	Positive		Prednisone, Pentasa
Jewish	not A	B	Positive	Active Mild-Moderate	Asacol, 6-MP
Jewish	A	A	negative	Inactive	6-MP, Asacol, Remicade every 8 wks
Jewish	A	A	negative		Sulfasalazine
Jewish	A	A	negative		Pentasa
Jewish	A	A	negative	Active	AZA
Jewish	A	A	negative	Active Mildly	6-MP, Asacol
Jewish	A	A	negative		Pentasa
Jewish	A	A	negative		Prednisone
Jewish	A	A	negative		6-MP
Jewish	A	A	negative		Imuran, asulfidine
Jewish	A	A	negative		Asacol
Jewish	B	B	negative		None
Jewish	B	B	negative	Active	Entocort, Colazal, Canasa supp.
Jewish	B	B	negative		None
Jewish	B	B	negative		Asathioprine
Jewish	not A	B	negative	Active Mildly	Prednisone, 6-MP
Jewish	not A	B	negative		Pentasa
Jewish	not A	B	negative		Purinethol
non Jewish	A	A	Positive	Active	None (Digest-tinol, aloe-based)
non Jewish	A	A	Positive	Active Mildly	Imuran, Pentasa
non Jewish	A	A	Positive		None
non Jewish	A	A	Positive		Prednisone, Cipro, Xifaxin
non Jewish	A	A	Positive	Inactive	None
non Jewish	B	B	Positive	Inactive	Prednisone, Pentasa, Xifaxan
non Jewish	B	B	Positive	Inactive bordering active	6-MP, Pentasa, Remicade
non Jewish	B	B	Positive		Colazal
non Jewish	B	B	Positive	Active	6-MP, Pentasa, Thalidomide
non Jewish	not A	B	Positive		Entocort, 6-MP
non Jewish	not A	B	Positive		Remicade
non Jewish	A	A	negative	Inactive	6-MP
non Jewish	A	A	negative	Active Mildly	Asacol, Remicade
non Jewish	A	A	negative	Active	Humira
non Jewish	A	A	negative	Active	Cansa supp.
non Jewish	A	A	negative	Active	Asacol, 6-MP, Remicade
non Jewish	A	A	negative	Inactive	AZA
non Jewish	A	A	negative	Inactive	Asacol
non Jewish	A	A	negative	Inactive	Prednisone
non Jewish	A	A	negative	Inactive	Humira; Cipro, Flagyl
non Jewish	A	A	negative	Active Mildly	None (other than curcumin, vitamins)
non Jewish	B	B	negative	Active	Prednisone, Humira
non Jewish	not A	B	negative	Active	Remicade
non Jewish	B	B	negative	Active Mildly	Pentasa, Remicade
non Jewish	B	B	negative	Active	6-MP, Pentasa, Entocort
non Jewish	not A	B	negative	Inactive	6-MP
non Jewish	not A	B	negative	Active	Pentasa, Curcumin, Probiotics
non Jewish	not A	B	negative	Active	Humira
non Jewish	not A	B	negative	Active Mildly	Humira
non Jewish	B	B	negative	Inactive	Remicade; prednisone
non Jewish	B	B	negative	Active Mildly	Asacol, Prednisone
non Jewish	not A	B	negative	Active Mildly	6-MP, Asacol
non Jewish	B	B	negative	Active Mildly	Remicade

### TNFSF15 genotyping and haplotype assignment

**Figure 1:**
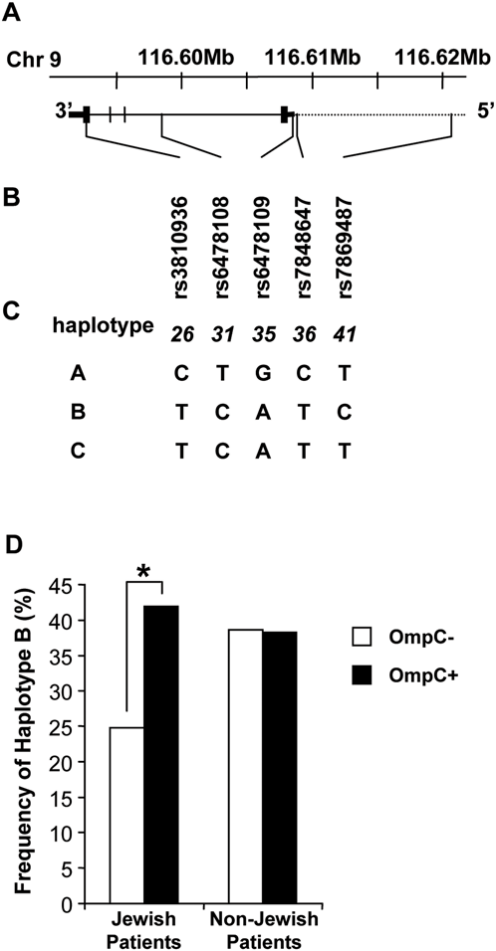
*TL1A (TNFSF15)* gene and haplotype structure and association with Crohn's Disease. (A) The *TNFSF15* gene is on the reverse strand of NCBI human genome build 36 at the position shown on *chromosome 9* but is shown 5′ to 3′ in keeping with the previous reports. The dotted line represents genomic sequence, solid line the gene sequence, thin bars untranslated regions, and thick bars the exons. (B) Single nucleotide polymorphisms (SNPs) genotyped for this study are listed with their relative positions along the gene. (C) Definition of haplotypes assigned in this study. The nucleotide assignments follow the previous report and correspond to the sense strand of the gene. The numbers 26, 31, 35, 36, and 41 refer to previous reports [12]. (D) Frequency of CD patients as a function of OmpC status for TL1A haplotype B for Jewish and non-Jewish CD patients, respectively (P≤0.001).

Single nucleotide polymorphisms (SNPs) *rs3810936, rs6478108, rs6478109, rs7848647*, and *rs7869487* were genotyped using either Illumina Golden Gate technology [27], [28] or ABI TaqMan MGB technology [29], [30] following the manufacturer's protocols (Illumina, San Diego, CA; ABI, Foster City, CA). Assays for these SNPs are available to other researchers through ABI as follows: *rs3810936, hCV363308; rs6478108, hCV170492; rs6478109, hCV1305297; rs7848647, hCV11277159;* and *rs7869487, hCV11277149*. Consistency between the two methods has been confirmed in our laboratory by genotyping over 200 samples with both methods. Details of the positions of these SNPs with respect to the *TNFSF15* gene and the haplotypes from the individual SNPs are shown in Figure 1. While redundancy in the 5 SNPs may be apparent from Figure 1, all 5 SNPs are retained in this study in order to (1) distinguish the major haplotypes from minor haplotypes with low frequencies (data not shown) and (2) retain the haplotype terminology of the previous reports [12], [13], [15]. Haplotypes were assigned from the individual SNP data using Phase v2 and the probability of assignment was greater than 95%. Association of haplotypes with the presence of antibody per subject was tested by chi-square and the significance was estimated by permutation (Jewish OmpC−: N = 304, Jewish OmpC+: N = 196, non-Jewish OmpC−: N = 434, non-Jewish OmpC+: N = 234).

### Serological Analysis

Sera were analyzed for expression of anti-OmpC antibodies by ELISA as previously described [31].

### Isolation of CD14^+^ monocytes from patient's blood

Blood was obtained from patients after informed consent in accordance with procedures established by the Cedars-Sinai Institutional Review Board. Monocytes were isolated from PBMC as described previously [7].

### Cell culture and stimulation

Plate-bound, cross-linked human IgG (IC) was prepared as described previously [7]. Monocytes were incubated with IC for the indicated time-points.

### ELISA

TL1A was quantified in undiluted supernatants from stimulated cells using an ELISA and Ab developed at Teva Pharmaceutical USA as described elsewhere [7].

### Flow cytometry

Monocytes stimulated with IC or bacteria for 16 h were stained with the TL1A Ab as described previously [7]. Cells were analyzed on a CyAn™ ADP flow cytometer (Dako Cytomation, Carpinteria, CA) and analyzed with the Summit 4.1 software package (Dako).

### Statistics

Statistical significance between groups was determined by Student's *t* test. A value of *p*≤0.05 was considered to be statistically significant.

## Results

### Association of *TNFSF15 (TL1A) haplotype B* and the serum expression of anti-OmpC in Jewish Crohn's disease patients

Since the expression of anti-OmpC in CD patients is a familial trait and has been associated with severe CD [23], [24], [32], we tested the association between *TNFSF15 haplotype B* and the serum expression of anti-OmpC in Ashkenazi Jewish and non-Jewish CD patients (Figure 1A–D). *TNFSF15 haplotypes* were assigned from individual SNP data using PHASE v2 with a probability greater than 95% (see Figure 1A–C for details of SNPs and haplotypes). In non-Jewish CD subjects, there was no difference in the frequency of *haplotype B* between OmpC positive and OmpC negative subjects (OmpC negative 38.6%, OmpC positive 38.3%; Figure 1D). The frequency of haplotype B in either group was similar to that previously observed for all CD in non-Jewish subjects [15]. In contrast, for Jewish CD subjects the frequency of *haplotype B* was significantly higher in OmpC positive subjects compared with OmpC negative subjects (Figure 1 D) (24.9% OmpC negative, 41.9% OmpC positive, P≤0.001). The frequency of *haplotype B* in OmpC negative subjects was lower and in OmpC positive subjects was higher than that previously observed for all CD in Jewish subjects (32%) [15] (Figure 1D). In addition, the level of anti-OmpC expression was higher in *TNFSF15 (TL1A) haplotype B* Jewish subjects (the median level of anti-OmpC was 23.9 ELISA units in Jewish CD patients with haplotype B compared with 14.9 in Jewish CD patients without *haplotype B*, P wilcoxon test ≤0.001). The frequency of OmpC seropositivity in Jewish CD patients with *haplotype B* is 52%, and 36% in non-Jewish CD patients with *haplotype B*. We did not observe an association between other CD-associated antibodies to microbial antigens (e.g. anti-Cbir1 antibodies, anti-I2 antibodies, anti-ASCA antibodies) and differences in *haplotype B* frequency in Jewish or non-Jewish CD patients (data not shown).

### *TNFSF15 (TL1A) haplotypes* in Jewish IBD patients results in differential TL1A expression in monocytes stimulated with IC

**Figure 2:**
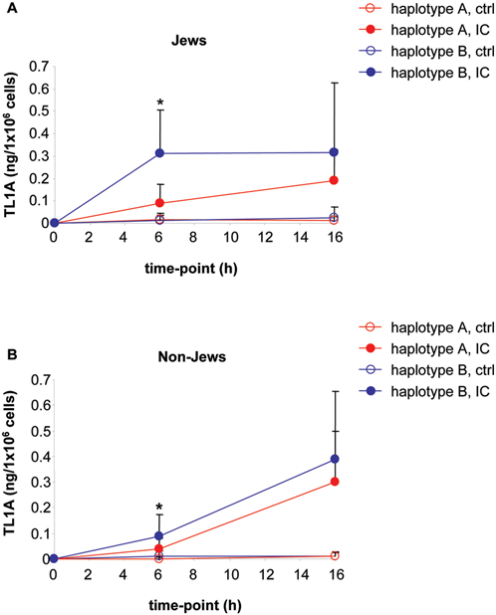
The *B* risk haplotype is associated with increased TL1A secretion in CD patients. Monocytes from Jewish and non-Jewish patients with A, or B haplotypes were stimulated with IC for 6 or 16 h. Cell culture supernatants were analyzed for TL1A secretion by ELISA. (A) Monocytes from Jewish patients with A or B haplotypes were stimulated with IC for 6 or 16 h (n = 15 and 12 for *haplotype A* and *haplotype B*, respectively, *, P≤0.002). All data represent the mean±SD. (B) Monocytes from non-Jewish patients with *A* or *B haplotypes* were stimulated with IC for 6 or 16 h (n = 13 and 15 for *haplotype A* and *haplotype B*, respectively, *, P≤0.05).

To determine if the different *TNFSF15 (TL1A) haplotypes* associated with CD in Jewish patients correlate with functional consequences in TL1A expression, we isolated CD14+ monocytes from Jewish and non-Jewish CD patients with *haplotypes A* or *B*, and stimulated them with immune complexes (IC), a known inducer of TL1A gene expression [7]. We observed strong induction of TL1A secretion in response to IC in Jewish *haplotype B* carriers at 6 h of stimulation. Compared to Jewish *haplotype A* carriers, the secretion was significantly increased at 6 h in *haplotype B* carriers (Figure 2A). At 16 h we also observed an increase, although not significant (p = 0.22), in secretion of TL1A in *haplotype B* compared to *haplotype A* Jewish patients (Figure 2A). In non-Jewish CD patients we also observed a significant difference between TL1A secretion in *haplotypes A* and *B* at 6 h of IC stimulation (Figure 2B). *Haplotype B* carriers secreted significantly more TL1A at 6 h compared to *haplotype A* carriers. At 16 h the difference in TL1A secretion between the haplotypes were not significant (p = 0.29), although we observed a higher secretion in *haplotype B* non-Jewish patients. To determine if there are ethnic differences in expression of TL1A we compared Jewish and non-Jewish *haplotype B* carriers. Jewish *haplotype B* carriers secreted significant more TL1A at 6 h compared to non-Jewish *haplotype B* (p = 0.002). At 16 h we did not observe any differences in the IC-induced secretion of TL1A (p = 0.49). Next, we compared the TL1A secretion in Jewish and non-Jewish *haplotype A* carriers. There were no differences in the IC-induced secretion of TL1A in Jewish or non-Jewish CD *haplotype A* patients at 6 or 16 h (p = 0.07 and 0.09, respectively).

**Figure 3:**
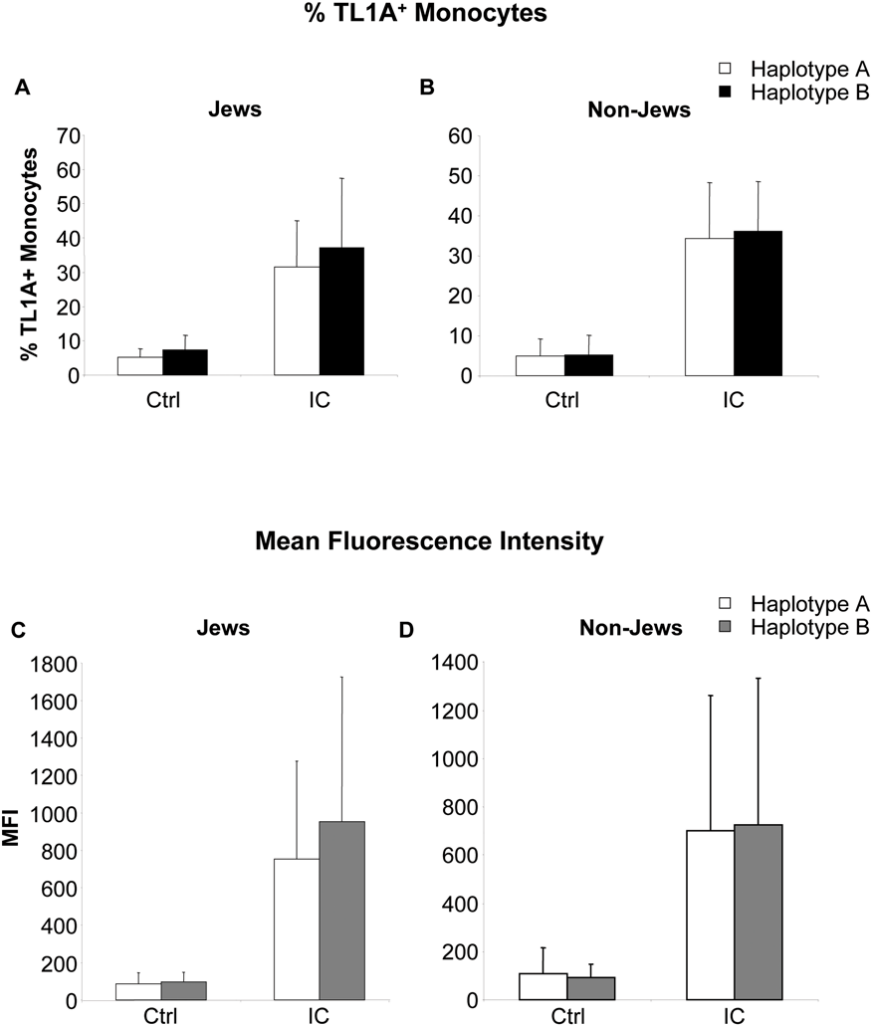
*Haplotype A* and *B* have similar TL1A surface expressions in Jewish and non-Jewish CD patients. (A) Monocytes from Jewish patients with *A* or *B haplotypes* were stimulated with IC for 16 h. Monocytes were stained with an anti-TL1A antibody and analyzed by flow cytometry. Data are presented as % of TL1A^+^ monocytes (n = 11 and 9 for *haplotype A* and *haplotype B*, respectively, P = 0.19 and P = 0.49 for control and IC, respectively). All data represent the mean±SD. (B) Monocytes from non-Jewish patients with *A* or *B haplotypes* were stimulated with IC for 16 h. Data are presented as % of TL1A^+^ monocytes (n = 14 and 16 for *haplotype A* and *haplotype B*, respectively, P = 0.83 and P = 0.72 for control and IC, respectively). (C) Monocytes from Jewish patients with *A* or *B haplotypes* were stimulated with IC for 16 h. Data are presented as mean fluorescence intensity (MFI) (n = 11 and 9 for *haplotype A* and *haplotype B*, respectively, P = 0.66 and P = 0.52 for control and IC, respectively). (D) Monocytes from non-Jewish patients with *A* or *B haplotypes* were stimulated with IC for 16 h. Data are presented as mean fluorescence intensity (MFI) (n = 14 and 16 for *haplotype A* and *haplotype B*, respectively, P = 0.56 and P = 0.91 for control and IC, respectively).

To determine if the observed differences in TL1A secretion also relate to differences in surface TL1A expression, we determined the expression of membrane TL1A as measured by the percentage of TL1A^+^ monocytes and mean fluorescence intensity (MFI) after IC stimulation (Figure 3). We did not observe any significant differences in the percentage of control or IC-stimulated TL1A^+^ monocytes in Jewish *haplotype B* carriers compared to *haplotype A* carriers (Figure 3A, p = 0.19 and p = 0.49 for control and IC stimulation, respectively). Furthermore, we also did not observe any significant differences in the percentage of control or IC-stimulated TL1A^+^ monocytes in non- Jewish *haplotype B* carriers compared to *haplotype A* carriers (Figure 3B, p = 0.83 and p = 0.72 for control and IC stimulation, respectively). When we analyzed MFI of TL1A staining we observed an increase in MFI in Jewish *haplotype B* compared to *haplotype A* carriers, although this increase did not reach significance (Figure 3C, p = 0.66 and p = 0.52 for control and IC stimulation, respectively). We did not observe any differences in MFI in non-Jewish *haplotype B* compared to *haplotype A* carriers (Figure 3D, p = 0.56 and p = 0.91 for control and IC stimulation, respectively). In summary, Jewish and non-Jewish *haplotype B* carriers secrete significantly more TL1A in response to IC compared to *haplotype A* carriers at 6 h (Table 2). Furthermore, Jewish *haplotype B* carriers secrete significantly more TL1A compared to non-Jewish *haplotype B* carriers (Figure 2C). In contrast, we did not observe any significant differences in TL1A surface expression in patients with different haplotypes in either Jewish or non-Jewish CD patients (Table 2).

**Table 2:** Summary of significant differences for TL1A *haplotypes A* and *B* in CD patients stratified into Jewish and Non-Jewish

	Jews	Non-Jews
**Soluble TL1A**	IC stimulation	IC stimulation
	haplotype B >>> haplotype A**	haplotype B > haplotype A*

* , *p*≤0.05

** , *p*≤0.005

### *TNFSF15 (TL1A)* haplotypes in Jewish OmpC+ IBD patients result in differential TL1A expression in monocytes stimulated with IC

**Figure 4:**
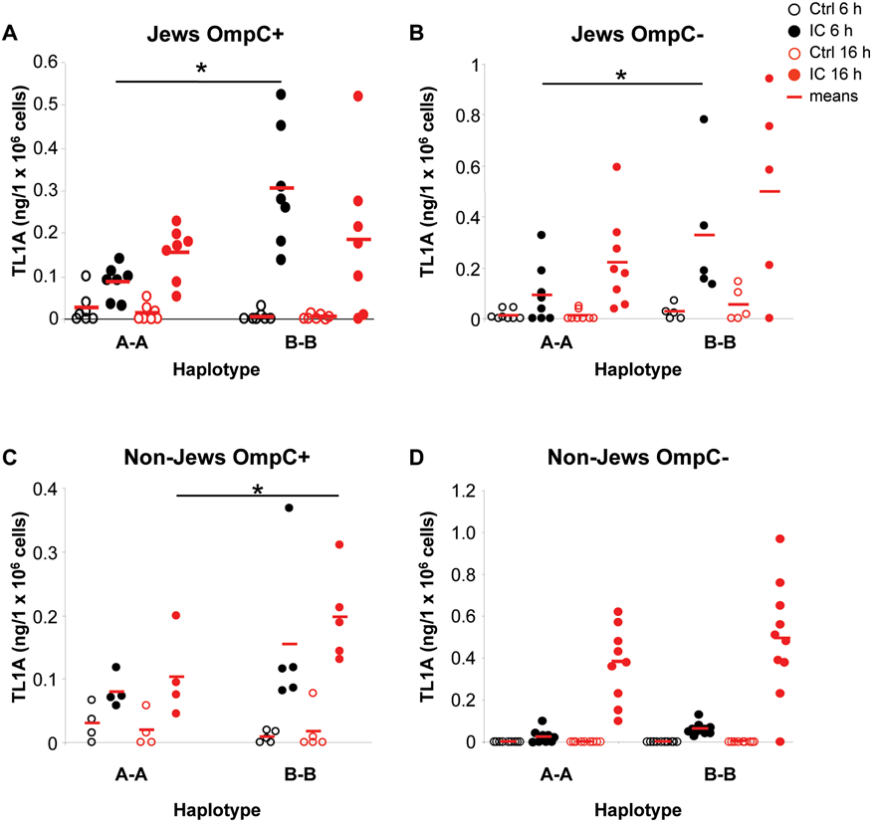
The *B* risk haplotype is associated with increased TL1A secretion in CD patient subgroups. (A) Monocytes from Jewish OmpC+ patients homozygous for *haplotypes A* or *B* were stimulated with IC for 6 or 16 h (n = 7 for *haplotype A* and *haplotype B*, *, P≤0.001). Cell culture supernatants were analyzed for TL1A by ELISA. (B) Monocytes from Jewish OmpC− patients homozygous for *haplotype A* or *haplotype B* were stimulated with IC for 6 or 16 h (n = 8 and 5 for *haplotype A* and *haplotype B*, respectively, *, P≤0.05). (C) Monocytes from non-Jewish OmpC+ patients with *A* or *B* haplotypes were stimulated with IC for 6 or 16 h (n = 4 and 5 for *haplotype A* and *haplotype B*, respectively, *, P≤0.05). (D) Monocytes from non-Jewish OmpC− patients with *A* or *B haplotypes* were stimulated with IC for 6 or 16 h (n = 9 and 10 for *A* and *B*, respectively).

Since we observed a risk association for TL1A *haplotype B* in Jewish CD patients, and this association was accentuated in Jewish OmpC+ patients, we subdivided the Jewish patients into OmpC+ and OmpC− patients and analyzed TL1A secretion and membrane expression for *haplotypes A* and *B*. We observed a significant increase in TL1A secretion in patients carrying the *B haplotype* compared to *A* carriers in Jewish OmpC+ patients in response to IC (Figure 4A). Differences in TL1A secretion were most prominent at 6 h post stimulation compared to 16 h (Figure 4A, p = 0.34). We observed a TL1A response in Jewish OmpC+ patients heterozygous for *haplotype A* and *B* that was intermediate between that observed for patients carrying the *A* and *B haplotype*, respectively (data not shown). Jewish OmpC− patients carrying the *B haplotype* also secreted higher levels of TL1A in response to IC than *A* carriers at 6 h post stimulation (Figure 4B, p = 0.054 for 16 h IC stimulation), while non-Jewish OmpC+ patients with the *B haplotype* secreted higher levels of TL1A at 16 h post stimulation (Figure 4C, p = 0.14 for 6 h IC stimulation), suggesting different kinetics in the TL1A secretion in Jewish vs. non-Jewish CD patients in response to FcγR signaling. We did not observe any differences in TL1A secretion in non-Jewish OmpC− patients with the *B haplotype* compared to patients with *haplotype A* (Figure 4D, p = 0.21 for 16 h IC stimulation).

**Figure 5:**
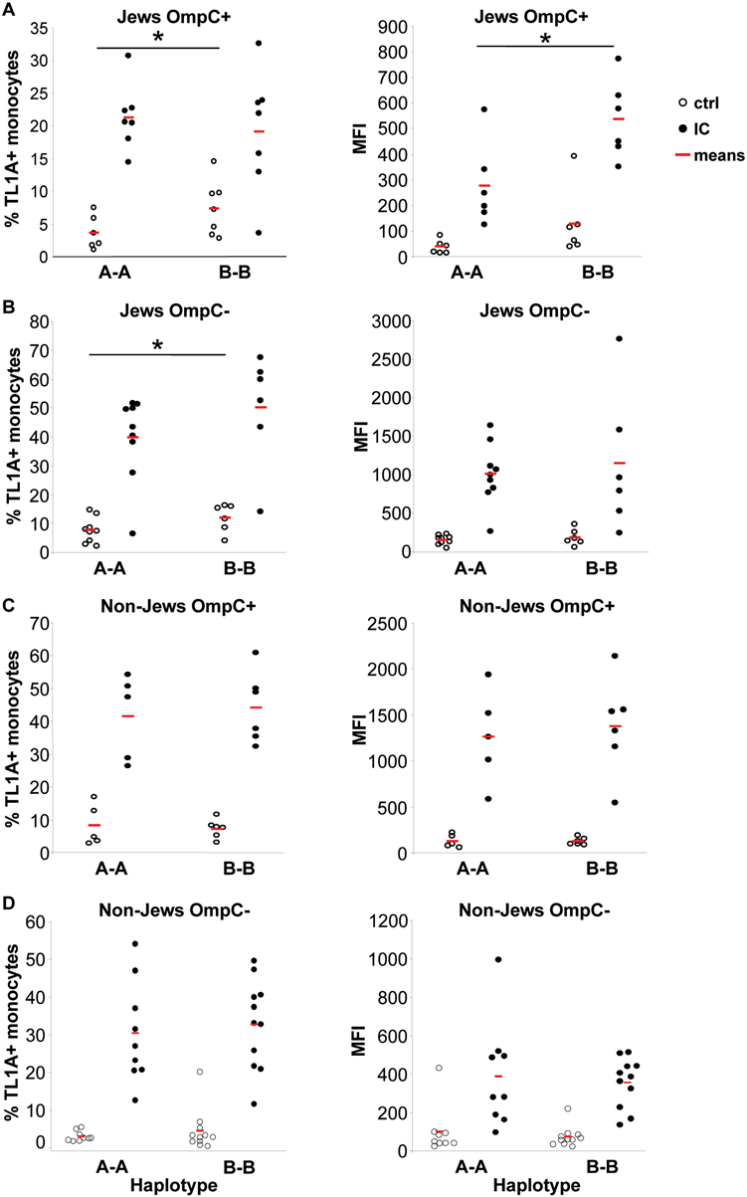
The *B* risk haplotype is associated with an increase in the number of TL1A+ peripheral monocytes in Jewish but not in non-Jewish CD patients. (A) Monocytes from Jewish OmpC+ patients with *A* or *B haplotypes* were stimulated with IC for 16 h. Monocytes were stained with an anti-TL1A antibody and analyzed by flow cytometry. Data are presented as % of TL1A^+^ monocytes (left panel) or mean fluorescence intensity (MFI) (right panel) (n = 6, or 7 for *haplotype A* or *haplotype B*, respectively, *, P≤0.05 for % TL1A^+^; *, P≤0.02 for MFI). (B) Monocytes from Jewish OmpC− patients with *A* or *B haplotypes* were stimulated with IC for 16 h. Data are presented as % of TL1A^+^ monocytes (left panel) or MFI (right panel). (n = 9 or 6 for *haplotype A* or *haplotype B*, respectively, *, P≤0.05) (C) Monocytes from non-Jewish OmpC+ patients with *A* or *B haplotypes* were stimulated with IC for 16 h. Data are presented as % of TL1A^+^ monocytes (left panel) or MFI (right panel). (n = 5 or 6 for *haplotype A* or *haplotype B*, respectively). (D) Monocytes from non-Jewish OmpC− patients with *A* or *B haplotypes* were stimulated with IC for 16 h. Data are presented as % of TL1A^+^ monocytes (left panel) or MFI (right panel). (n = 9 or 11 for *haplotype A* or *haplotype B*, respectively).

Next, we analyzed the surface expression of TL1A in Jewish OmpC+ patients with different haplotypes by flow cytometry (Figure 5). We observed a significant increase in the proportion of TL1A^+^ monocytes in untreated monocytes from Jewish patients carrying the *B haplotype* compared to patients with the *A haplotype* in Jewish patients regardless of their OmpC status (Figure 5A, B). Similar differences were not seen in non-Jewish patients (Figure 5C, D, p = 0.37 and p = 0.43 for non-Jewish OmpC+ and non-Jewish OmpC− patients, respectively). Surprisingly, we could not observe any differences in the percentage of monocytes expressing TL1A stimulated with IC between different *haplotypes* (Figure 5A, B, p = 0.31). However, the mean fluorescence intensity (MFI) was significantly increased in IC stimulated monocytes in Jewish OmpC+ patients carrying the *B haplotype* compared to *A* carriers (Figure 5A) but not in Jewish OmpC− patients (Figure 5B, p = 0.35). We did not observe any significant differences in surface expression of TL1A in untreated or IC stimulated monocytes between *A* and *B* carriers in non-Jewish patients (Figure 5C, D, p = 0.35 and p = 0.36 for IC-stimulated monocytes from non-Jewish OmpC+ patients, % TL1A+ monocytes and MFI, respectively, and p = 0.68 and p = 0.74 for IC-stimulated monocytes from non-Jewish OmpC+ patients, % TL1A+ monocytes and MFI, respectively). In summary, Jewish OmpC+ and OmpC− *haplotype B* carriers secrete significantly more TL1A in response to IC compared to *haplotype A* carriers (Table 3). Furthermore, the proportion of TL1A^+^ monocytes in untreated monocytes is significantly increased in Jewish OmpC+ and OmpC− *haplotype B* carriers compared to Jewish OmpC+ and OmpC− *haplotype A* carriers. Additionally, the MFI of TL1A membrane expression was significantly increased in IC stimulated monocytes in Jewish OmpC+ patients with the *B haplotype* compared to patients with the *A haplotype* (Table 3).

**Table 3:** Summary of significant differences for TL1A *haplotypes A* and *B* in CD patients sub-stratified into Jewish OmpC+/OmpC− and non-Jewish OmpC+/OmpC−

	Jews OmpC+	Jews OmpC−	Non-Jews OmpC+	Non-Jews OmpC−
**Soluble TL1A**	IC stimulation	IC stimulation	IC stimulation	IC stimulation
	haplotype B > haplotype A***	haplotype B > haplotype A*	haplotype B > haplotype A*	—
**Membrane TL1A**				
**(** ***% TL1A+ Monocytes)***	baseline	baseline	baseline	baseline
	haplotype B > haplotype A *	haplotype B > haplotype A *	—	—
***(MFI)***	IC stimulation	IC stimulation	IC stimulation	IC stimulation
	haplotype B > haplotype A **	—	—	—

* , *p*≤0.05

** , *p*≤0.01

*** , *p*≤0.001

## Discussion

Our findings demonstrate a direct association between genetic variation in the TL1A gene *TNFSF15*, and the induction of TL1A in Jewish, and particularly in OmpC+ Jewish CD patients. Patients carrying the *TNFSF15* (*TL1A*) risk *haplotype B* have a significantly increased expression of membrane and soluble TL1A in response to IC. In the current era of finding genetic associations with disease using genome-wide association studies, our report also illustrates that detailed molecular phenotypic studies of patients with particular haplotypes will lead to the discovery of important functional differences that occur as a result of the different haplotypes.

We have previously reported that *TNFSF15* (*TL1A*) *haplotype B* is associated with a more severe disease phenotype in Jewish patients, as these individuals have a higher frequency of small bowel surgery [15]. Furthermore, *TNFSF15 haplotype B* is associated with increased risk of CD in Ashkenazi Jews (32% in CD vs. 26% in controls) but with a decreased risk in non-Jewish CD patients (39% in CD vs. 50% in controls) [15]. Here, we extend our findings and demonstrate that Jewish OmpC+ CD patients (a group characterized by more severe disease) have a further increased frequency of *haplotype B* (also a group characterized by more severe disease) (42% CD OmpC+ vs. 25% CD OmpC−) suggesting that an antibody titer for the *E. coli* outer membrane porin C (OmpC) increases the risk of CD in Jewish patients carrying the *B haplotype*. We observed that this hierarchical relationship of *TL1A* risk *haplotype B* and OmpC titers in Jewish CD patients also applies to the functional response, e.g. the immune responses to FcγR signaling in Jewish CD patients. In both Jewish and non-Jewish CD we observed a significant increase of TL1A secretion in *haplotype B* compared to *haplotype A*. However, we observed an earlier onset of TL1A secretion and an overall greater amount of TL1A secretion in Jewish patients with *haplotype B* compared to non-Jewish *haplotype B* carriers, suggesting different kinetics in the induction of TL1A expression in Jewish vs. non-Jewish patients carrying haplotype B (see Table 2 for data summary). When we further sub-stratified Jewish and non-Jewish patients into patient groups with or without positive serology for OmpC antibodies, this association of *TL1A haplotype B* and increased TL1A expression was further accentuated in Jewish OmpC+ patients. Jewish OmpC+ patients with *haplotype B* have increased secretion of soluble and enhanced expression of membrane TL1A in response to IC, as well as increased baseline membrane TL1A expression, when compared to *haplotype A* (see Table 3 for data summary). On the other hand Jewish OmpC− patients with *haplotype B* have increased secretion of soluble TL1A in response to IC, and increased baseline membrane TL1A expression, but TL1A and membrane TL1A in response to IC are not significantly different when compared to *haplotype A*. In non-Jewish OmpC+ patients with *haplotype B*, we only observed an increased secretion of soluble TL1A in response to IC, but with a distinct and delayed kinetics compared to Jewish OmpC+ and OmpC− patients of the same haplotype. The higher baseline expression of membrane TL1A on monocytes that we observed in Jewish but not non-Jewish CD patients carrying *haplotype B* might suggest a higher capacity of TL1A dependent T cell activation in these patients [5], [7].

Interestingly, we observed that the percentages of monocytes with TL1A surface expression being approximately twice as high in OmpC− Jewish patients compared to OmpC+ patients for either haplotype when stimulated with IC (Figures 5A, B). We analyzed the TL1A surface expression of monocytes in response to IC stimulation at a 16 h time-point. We have demonstrated that monocytes from Jewish OmpC+ and OmpC− patients have a faster induction of TL1A secretion compared to non-Jewish patients (Figures 2, 4). We observed maximal induction of TL1A secretion in Jewish OmpC+ patients at 6 h and a decline of TL1A secretion at 16 h. However, in Jewish OmpC− patients we observed a further increase of TL1A secretion at 16 h (Figure 4 A and B). The mechanism for the differential induction in Jewish OmpC+ vs. Jewish OmpC− and Jewish vs. non-Jewish patients is currently unknown. The differences in surface expression in Jewish OmpC+ vs. OmpC− patients might therefore just reflect the differences in cleavage-kinetics of TL1A from the membrane due to higher turn-over of TL1A in Jewish OmpC+ patients.

We previously published that *TNFSF15 haplotype B* is associated with increased risk of CD in Ashkenazi Jews but with a decreased risk in non-Jewish CD patients [15]. To our surprise, we did not observe a decrease in TL1A expression in *haplotype B* carriers in non-Jewish patients compared to *haplotype A* carriers as one would expect (Figures 2B, 3B, 3D). However, from the data presented here we are concluding that Jewish CD patients demonstrate a faster TL1A induction than non-Jewish patients. We observed an increase of TL1A secretion at 16 h compared to 6 h post IC-stimulation in non-Jewish patients suggesting a maximal secretion at 16 h. However, we did not analyze the TL1A secretion beyond the 16 h time-point and it might be possible that in non-Jewish patients the maximal secretion of TL1A is reached beyond 16 h. Therefore, it cannot be excluded that a difference between TL1A secretion in haplotype A and B might occur at a later time-point in non-Jewish patients. Furthermore, based on our data we hypothesize that TL1A is a CD severity more importantly than susceptibility gene. We have previously demonstrated that *TNFSF15 haplotype B* is associated with increased risk of CD in Jews but with a decreased risk in non-Jewish CD patients [15]. However, when we analyzed haplotype association with disease severity, as measured by clinical parameters (fibrostenosis, internal perforation, perianal penetration, small bowel surgeries), no association was observed between haplotypes in non-Jewish patients but association was observed in Jewish CD patients carrying haplotype B [15]. These data are consistent with our findings of no significant differences in TL1A expression in non-Jewish patients.

The *TNFSF15* genetic variation and TL1A functional changes observed here are of importance in the context of the recent demonstration that the *IL23R* gene is a major determinant of CD susceptibility [14], [33], [34], [35], [36], [37], [38], [39]. In particular, we have been able to demonstrate that TL1A synergizes with IL-23 to induce IL-17 production by CD4+ T cells in a mouse model of chronic colitis [8]. Furthermore, these observations are consistent with the role of TL1A during the development of chronic colitis in mice. We have been able to demonstrate that administration of neutralizing TL1A antibodies not only prevents but also treats established chronic colitis, likely by directly blocking IFN-γ production by T_H_1 cells and IL-17 and IL-6 production from T_H_17 cells, and also by blocking the enhancing effect of TL1A/IL-12 and TL1A/IL-23 on these CD4^+^ T cell subsets [8]. These data support the notion that TL1A expression is increased during chronic mucosal inflammation and that leads to further activation of TH1 and TH17 cells.

In this study, we demonstrated that there is a direct association between genetic variants of the *TL1A* gene and the induction of TL1A in Jewish CD patients. Since the induction of TL1A gene expression seems to be restricted to stimulation by FcγR signaling and/or enteric bacteria [7], [40], [41], blockage of TL1A might have therapeutic benefit in IBD patients without markedly increasing the susceptibility to infection. Defining CD patients by ethnicity, serotyping, and genotyping, will allow us to identify patients with the potential for higher TL1A response to immune complexes and commensal bacteria and hence may well identify those CD patients that would respond best to therapeutic blockade of TL1A function.
